# Estimation of maximum intraventricular pressure: a three-dimensional fluid–structure interaction model

**DOI:** 10.1186/1475-925X-12-122

**Published:** 2013-11-22

**Authors:** Hamidreza Ghasemi Bahraseman, Kamran Hassani, Arezoo khosravi, Mahdi Navidbakhsh, Daniel M Espino, Davood Kazemi-Saleh, Naser Fatourayee

**Affiliations:** 1 Department of Biomechanics, Science and Research Branch, Islamic Azad University, Tehran, Iran; 2 Atherosclerosis research center, Tehran, Iran; 3 Department of Mechanical Engineering, Iran University of Science and Technology, Tehran, Iran; 4 School of Mechanical Engineering, University of Birmingham, Birmingham, UK; 5 Department of Biomedical Engineering, Amirkabir University, Tehran, Iran

## Abstract

**Background:**

The aim of this study was to propose a method to estimate the maximum pressure in the left ventricle (MPLV) for a healthy subject, based on cardiac outputs measured by echo-Doppler (non-invasive) and catheterization (invasive) techniques at rest and during exercise.

**Methods:**

Blood flow through aortic valve was measured by Doppler flow echocardiography. Aortic valve geometry was calculated by echocardiographic imaging. A Fluid–structure Interaction (FSI) simulation was performed, using an Arbitrary Lagrangian–Eulerian (ALE) mesh. Boundary conditions were defined as pressure loads on ventricular and aortic sides during ejection phase. The FSI simulation was used to determine a numerical relationship between the cardiac output to aortic diastolic and left ventricular pressures. This relationship enabled the prediction of pressure loads from cardiac outputs measured by invasive and non-invasive clinical methods.

**Results:**

Ventricular systolic pressure peak was calculated from cardiac output of Doppler, Fick oximetric and Thermodilution methods leading to a 22%, 18% and 24% increment throughout exercise, respectively. The mean gradients obtained from curves of ventricular systolic pressure based on Doppler, Fick oximetric and Thermodilution methods were 0.48, 0.41 and 0.56 mmHg/heart rate, respectively. Predicted Fick-MPLV differed by 4.7%, Thermodilution-MPLV by 30% and Doppler-MPLV by 12%, when compared to clinical reports.

**Conclusions:**

Preliminary results from one subject show results that are in the range of literature values. The method needs to be validated by further testing, including independent measurements of intraventricular pressure. Since flow depends on the pressure loads, measuring more accurate intraventricular pressures helps to understand the cardiac flow dynamics for better clinical diagnosis. Furthermore, the method is non-invasive, safe, cheap and more practical. As clinical Fick-measured values have been known to be more accurate, our Fick-based prediction could be the most applicable.

## Background

Cardiac disease is a major cause of death in industrialized countries, despite advances in prevention, diagnosis, and therapy [1]. Maximum pressure in the left ventricle (MPLV) assessment is among the most clinical measured for cardiac disease is important for disease recognition [2]. However, its measurement requires invasive techniques. Therefore, this study has assessed a Fluid–structure Interaction (FSI) method to predict MPLV and trans-aortic pressure, as a non-invasive alternative to current methods.

Invasive techniques used to measure MPLV include Fick oximetric and Thermodilution [3], catheterisation alone [4] or with echo-Doppler [5] but have associated risks [3]. Hence, non-invasive measures have been correlated to invasive MPLV measurements [6-8]. However, MPLV may vary with heart rate and/or exercise but few studies have investigated this effect [9]. This includes computational models (e.g. FSI, left intraventricular-impedance), which have so far neglected the exercise on intraventricular pressure gradients [10-12] despite the potential for inclusion of exercise modelling [13].

FSI simulations are overall well matched to cardiovascular modeling [14,15]. This method requires the use of an Arbitrary Lagrange-Euler (ALE) mesh to analyze both structural deformation and fluid flow; i.e. Computational Fluid Dynamics and Finite Element Analysis [16,17]. Recently, FSI has been used to investigate heart valves [18-25]. Previously, using two-dimensional geometry, we have measured the cardiac output and stroke volume for a healthy subject by coupling an echo-Doppler method with an FSI simulation at rest and during exercise. Particular attention was given to validating the model against measures of cardiac function that could be reliably calculated by applying clinical protocols, with varying exercise [13]. The effect of exercise on blood flow hemodynamics including the change of flow patterns across the aortic valve, vorticity, shear rate, stress and strain on the leaflets during exercise were also assessed [26]. In our previous studies pressures across the aorta were measured experimentally and applied to models. However, MPLV was not predicted.

The aim of this study is to propose and develop an FSI computational model capable of predicting MPLV for a healthy subject. The model uses the relationship of cardiac output to MPLV derived from invasive clinical cardiac output measurement. First, the relationship between Cardiac output and systolic ventricular pressure and systolic aortic pressure is derived, using a three-dimensional geometry. Christie et al. [27] obtained equations for Thermodilution cardiac output (COT) and Fick oximetric cardiac output (COF) to Doppler cardiac output (COD), in a clinical setting. Therefore, COT and COF were measured for the subject. Subsequently, MPLV was calculated noting to the numerical relationship between cardiac output, systolic ventricular pressure and systolic aortic pressure.

## Methods

### Combined clinical and numerical approach

#### Design of experiment

A healthy male, aged 33, with normal cardiovascular function had his hemodynamic data recorded while rest and exercise. Informed consent was acquired for the participant in line with accepted procedures approved by the Department of Cardiovascular Imaging (Shaheed Rajaei Cardiovascular, Medical and Research Center, Tehran, Iran).

#### Cardiovascular measurements

Hemodynamic data was assessed from maximal bicycle exercise tests and Doppler ECG.

**Figure 1:**
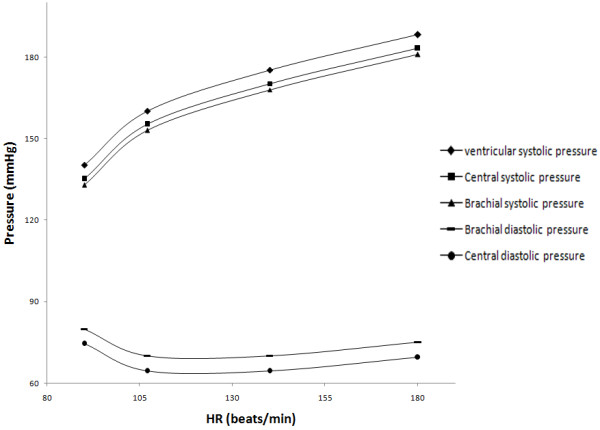
Interpolated curves for brachial, aortic and ventricular pressures.

Systolic and diastolic pressures of the brachial artery were measured and related to heart rate changes at rest and exercise (Figure 1). Equations 1 and 2 were used to determine the aortic pressure from brachial aortic pressure measurements. This relationship was previously determined by comparing brachial pressure (acquired by Oscillometry) to the aortic pressure acquired using an invasive method [28].

(1) Aorticsystolicpressure»Brachialsystolicpressure+2.25

(2) Aorticdiastolicpressure≈Brachialdiastolicpressure–5.45

where all pressures were measured in *mmHg*.

Left ventricular systolic pressure was derived from the calculated aortic systolic pressure. Previously, a pressure difference of around 5 mmHg was found between peak left ventricular systolic pressure and aortic systolic pressure, using catheterization [29]. The ejection times were derived from Doppler-flow imaging under B-mode.

#### Geometry

**Figure 2:**
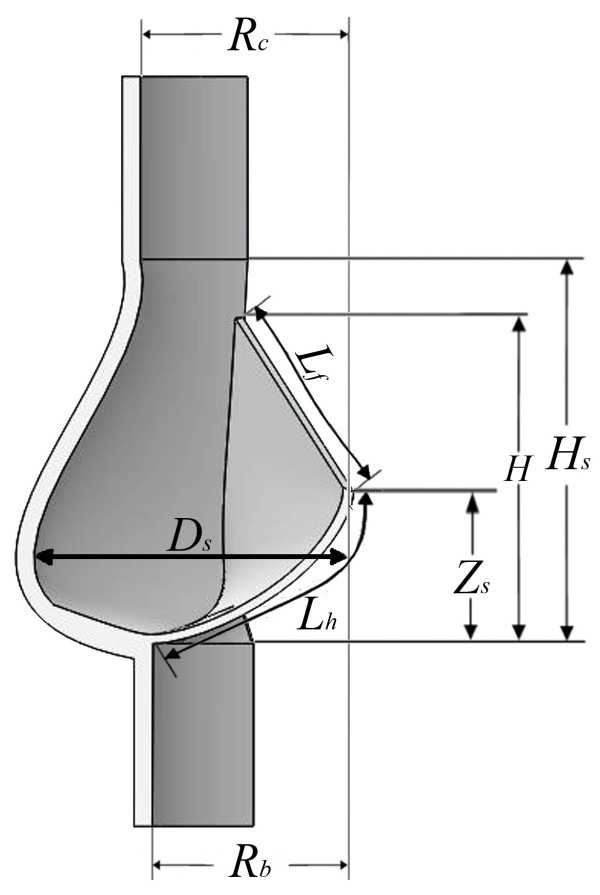
**The simulated aortic valve geometry.** An axisymmetric model was used with one-sixth of the valve represented. The top view was acquired using the assumed symmetry of the model.

A three-dimensional axisymetric model has been used with one-sixth of the valve geometry modelled (Figure 2; Table 1). Briefly, aortic valve geometry was obtained with respect to T-wave of ECG (maximum opening area). Diameters of the aortic valve annulus and the sinus valsalva were measured at the peak T-wave time using a resting para-sternal long-axis view. This data was used to generate the three-dimensional geometry (Figure 2) in Solidworks (Solidworks v2011, Dassault Systèmes SolidWorks Corp, France). In our model, leaflets were assumed to have a uniform thickness (0.6 mm).

**Table 1:** **Geometric parameters of the aortic valve as shown in Figure **2

**R**_**b**_	**R**_**c**_	**H**	**H**_**s**_	**L**_**h**_	**L**_**f**_	**D**_**s**_	**Z**_**s**_
**Radius of the base (mm)**	**Radius at the commissures (mm)**	**Valve height (mm)**	**Sinus height (mm)**	**Leaflet height (mm)**	**Leaflet free edge length (mm)**	**Sinus maximum radius (mm)**	**Sinus maximum radius location (mm)**
11.5	11.75	16.1	20.36	14	14.95	16.65	8.30

#### Fluid–structure interaction simulation

Valve cusps were considered to be isotropic, homogenous and to have a linear stress–strain relationship. This assumption has been used in other heart valve models [19,22,23,30]. Blood was assumed to be an incompressible and Newtonian fluid [15]. All material properties are provided in Table 2 and were obtained from the literature [31,32].

**Table 2:** Mechanical properties

**Viscosity (Pa.s)**	**Density (kg/m**^**3**^**)**	**Young’s modulus (N/m**^**2**^**)**	**Poisson ratio**
3.5 x 10^-3^	1056	6.885 x 10^6^	0.4999

**Figure 3:**
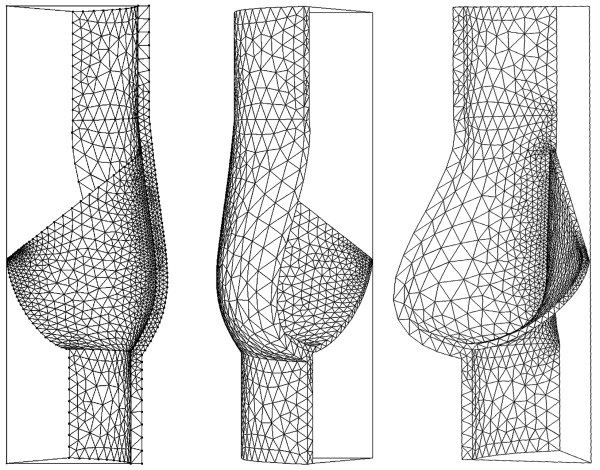
Generated mesh.

For fluid boundaries (Figure 2), pressure was applied at the inflow boundary of the aortic root at the left ventricular side. A moving ALE mesh was used which enabled the deformation of the fluid mesh to be tracked without the need for re-meshing [33]. Second order Lagrangian elements were used to define the mesh. The mesh contained a total of 87357 elements (Figure 3). The finite element analysis package Comsol Multi-physics (v4.2, Londen: Comsol Ltd.) was used to solve the FSI model under time dependent conditions [22,23,34]. The fluid velocity is coupled to the structural deformation while the valve is loaded by the fluid, this ensures simultaneous coupling [35-37].

### Hemodynamic measurements and relationships

Cardiac output was computed using Equation 3:

(3) Cardiacoutput=Strokevolume*Heartrate

where the stroke volume was calculated from ECG using Equation 4:

(4) Strokevolume=Velocity*Aorticarea

where the velocity integration was automatically obtained by tracing the Doppler flow from ultrasound imaging. The aortic area was calculated using Equation 5:

(5) $$\mathrm{Area}=\pi {\left(\frac{D}{2}\right)}^{2}$$

where ***D*** is the measured ascending aortic diameter after the sinotubular junction (Table 1).

**Figure 4:**
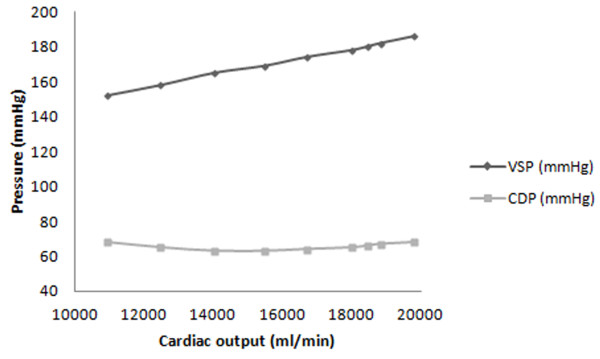
FSI prediction of aortic diastolic pressure’s change relative to heart rate based on Doppler method (ADPD), Fick oximetric method (ADPF), Thermodilution method (ADPT).

For FSI simulations, the aortic diastolic pressure’s change was obtained at each time step of the ejection period as shown in Figures 4. Equation 6, however, was used to determine the velocity integration (used to determine both stroke volume and cardiac output).

(6) $$\mathrm{Velocity}\;\mathrm{intergration}=\oint_{0}^{\text{Ejection time}}V.\mathrm{dt}$$

where ***V*** is the fluid-velocity through the outlet boundary. Stroke volume and cardiac output predicted from FSI simulations were compared to values determined by echo-Doppler. Note that the mean velocity for each heart rate was obtained using Equation 7.

(7) $$\mathrm{Velocity}\;\mathrm{mean}=\frac{\mathrm{Velocity}\;\mathrm{intergration}}{\mathrm{Ejection}\;\mathrm{time}}$$

## Results

**Figure 5:**
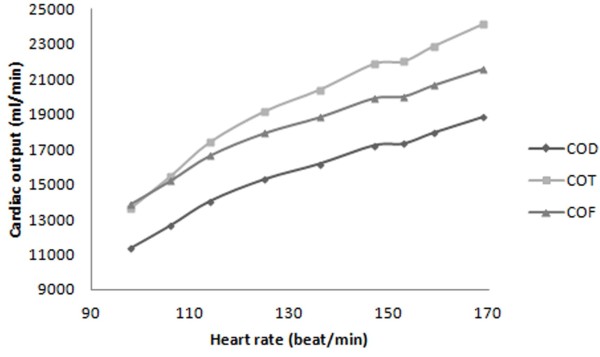
Numerically predicted ventricular systolic pressure (VSP) and Aortic diastolic pressure (ADP) relationship to cardiac output.

Doppler and numerical cardiac outputs are provided in Table 3. They used to calculate Left ventricular systolic pressure (VSP; Equation 8) and Aortic diastolic pressure (ADP; Equation 4) to the cardiac output predicted numerically (Figure 5 and Table 3):

(8) $$\mathrm{VSP}=-4.497\times 10^{-9}{\left(\mathrm{CO}\right)}^{2}-0.003868\left(\mathrm{CO}\right)+110.6;\left(R^{2}=0.9977\right)$$

(9) $$\mathrm{ADP}=2.56\times 10^{-7}{\left(\mathrm{CO}\right)}^{2}-0.007788\left(\mathrm{CO}\right)+122.3;\left(R^{2}=0.9674\right)$$

**Table 3:** Hemodynamic measurements

**Heart rate (bpm)**	**Ventricular systolic pressure (mmHg)**	**Aortic diastolic pressure (mmHg)**	**Doppler-cardiac output (ml/min)**	**Numerical-cardiac output (ml/min)**
98	152	68	11356	10916.97
106	158	65	12651	12478.27
114	165	63	14051	14031.32
125	169	63	15298	15487.93
136	174	64	16172	16686.83
147	178	65	17225	18012.27
153	180	66	17330	18445.6
159	182	67	17941	18844.08
169	186	68	18849	19817.15

Note that *CO* and *Hr* refer to cardiac output and heart rate, respectively.

A relationship between Doppler cardiac output and heart rate was obtained, using Table 3[38]:

(10) $$\mathrm{COD}=-0.7534{\left(\mathrm{Hr}\right)}^{2}-300.7\left(\mathrm{Hr}\right)+10710;\left(R^{2}=0.9934\right)$$

Christie et al. [27] obtained regression equations for the relationship between Thermodilution cardiac output (*COT*) and Fick oximetric cardiac output (*COF*) to Doppler cardiac output (COD), based on the data given from 15 subjects:

(11) $$\mathrm{COT}=1.41\left(\mathrm{COD}\right)-2394$$

(12) $$\mathrm{COF}=1.03\left(\mathrm{COD}\right)+2165$$

**Figure 6:**
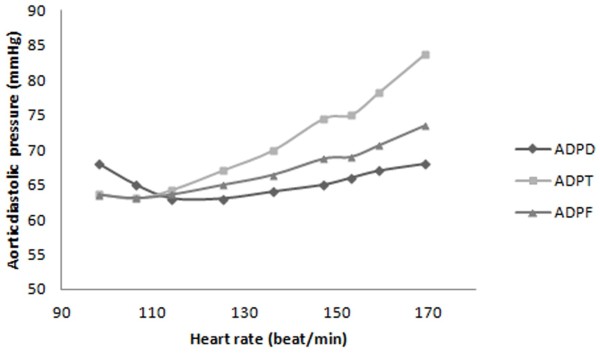
FSI prediction of cardiac output’s change relative to heart rate based on Doppler method (COD), Fick oximetric method (COF), Thermodilution method (COT).

Relationships between Fick oximetric (*COF*) and Thermodilution cardiac output (*COF*) relative to the heart rate (Figure 6) have been derived by combining above mentioned equations:

(13) $$\mathrm{COT}=-1.062{\left(\mathrm{Hr}\right)}^{2}+424\left(\mathrm{CO}\right)+17500;\left(R^{2}=0.9934\right)$$

(14) $$\mathrm{COF}=-0.776{\left(\mathrm{Hr}\right)}^{2}+309\left(\mathrm{Hr}\right)-8870;\left(R^{2}=0.9934\right)$$

**Figure 7:**
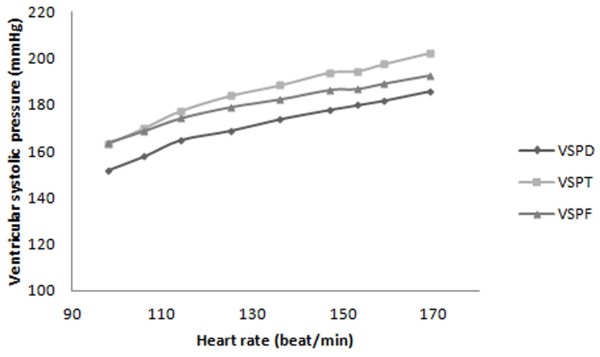
FSI prediction of ventricular systolic pressure’s change relative to heart rate based on Doppler method (VSPD), Fick oximetric method (VSPF), Thermodilution method (VSPT).

Variation in aortic diastolic (Figure 4) and left ventricular systolic (Figure 7) pressures with heart rate, have been derived from Thermodilution, Fick oximetric, and Doppler methods using Equations 8, 9, 10, 11, 12, 13 and 14. Both pressures rise with heart rate, despite differences in absolute values predicted using the different methods.

Aortic diastolic pressure, derived from Doppler based measurements, increased by 1% (0.7 mmHg) with increasing heart rate from 98 bpm to 169 bpm. This compares to 17% (10 mmHg) using Fick oximetry and 31.3% (19.9 mmHg) using thermodilution. The gradients of aortic diastolic pressure to heart rate were 0.04, 0.14 and 0.28 mmHg/heart rate measured using Doppler, Fick oximetric and Thermodilution, respectively.

The ventricular systolic pressure, predicted from the Doppler method, increased 22% (34 mmHg), with increasing heart rate from 98 bpm to 169 bpm (Figure 7). This increase was calculated to be 18% (28.9 mmHg) using Fick oximetry and 24% (39.6 mmHg) for Thermodilution. The gradients of ventricular systolic pressure to heart rate were 0.48, 0.41 and 0.56 (mmHg/heart rate) measured using Doppler, Fick oximetric and Thermodilution, respectively.

## Discussion

### Study findings

The study has combined an FSI model with hemodynamic measurements of the cardiac output from a healthy subject [13] and invasive clinical measurements [27] in order to estimation of maximum pressure in the left ventricles during exercise. Using a three-dimensional model, the method developed has potential for clinical application (see Initial insights in to clinical application & reliability section) and the obtained values show good agreement with the literature (see comparison to literature section). Moreover, the FSI model reliably predicted MPLV over a range of heart rates based on clinical measurement of cardiac outputs. MPLV was calculated by cardiac output of Doppler method, Fick oximetric and Thermodilution method which shows 22%, 18% and 24% increment during exercise, respectively.

### Initial insights in to clinical application & reliability

Regression analysis between echo-Doppler and FSI simulations resulted in a strong correlation (r = 0.998; Table 3) for cardiac output. Therefore, there was a strong correlation between the two mentioned methods, as clinical and computational techniques, with similar values were predicted. Predicting reliable intraventricular pressures is important in clinical diagnosis and treatment [2]. For instance, a recent commercial device to assess intraventricular pressure has a fluid-filled, balloon-tipped, catheter that is intended for insertion into the ventricle [39]. The balloon provides a closed system from which intraventricular pressure is determined. Its use is often limited to animal studies because of the risks involved with this invasive device.

Catheterization-Thermodilution is the current gold-standard for measuring intraventricular pressure [3]. It is an invasive procedure with potential risks such as heart failure, cardiac arrhythmia, and even death [3]. Moreover, Thermodilution exposes the patient and doctor to radiation. Exercising while catheterized results in a range of practical problems too, therefore, is not a common customary action. However, numerical methods enable estimation of cardiac function by non-invasive measurements during an exercise protocol. Therefore, the key-concern is the dependability of numerical methods when predicting MPLV while exercise.

### Comparison to literature

Following a literature search we have not found a previous comparable study that combined a clinical and numerical approach to predict MPLV during exercise. In our study, the patient specific MPLV were predicted at a range of heart rates induced by exercise for echo-Doppler, Thermodilution , and Fick oximetric methods. While the variation for MPLV from rest to peak of external work is established [40] this is the first study to use numerical methods to predict these values for an individual. Textbook MPLV gradients range from 35–51 mmHg/Heart rate for non-athletes, such as our subject, during the normal exercise cycle [40]. Our Thermodilution-based prediction lead to an overestimate of about 30%, our Fick oximetric-based prediction is underestimated by 4.7% and our Doppler prediction is overestimated by 12% when compared to standard textbook average values. Our results are in agreement with reports that Fick based methods provide more reliable measurements [41-45].

Textbook maximum systolic pressure for healthy left ventricles range from 250 to 300 mmHg, but varies widely among different subjects with heart strength and degree of heart stimulation by cardiac nerves [9]. MPLV measured through catheterization has ranged between 121 mmHg (at 75 bpm) to 210 mmHg (at 180 bpm) [9]. A study of healthy patients without valve abnormalities found the mean MPLV to be 121 mmHg (at 75 bpm, at rest) and 149 mmHg (at 108 bpm, during exercise).

### Limitations & future trends

The main limitations are that:

■ mechanical properties have been simplified and a constant single diameter has been used for the ascending aorta in the model;

■ statistical and generalized data are typically used for clinical assessment of hemodynamics but in our study only one subject was used for the initial development of a method for MPLV prediction.

Despite model limitations we presented excellent agreement with clinical measurements and the general literature [13]. A full three-dimensional model could result in more precise predictions, while, it would also increase the solution time (currently about 17 hours). This would hold disadvantages for clinical applications. Furthermore, a range of values for statistical comparison are not predictable without including model variability [23]. However, at this time, there is a tendency towards patient specific models [46], due to potential profits in aiding treatment/diagnosis for an individual. Prediction of intraventricular pressure could also be useful to construct more reliable heart valve prototypes [47].

## Conclusions

This study demonstrates the technical feasibility of combining a three-dimensional fluid- structure interaction model of the aortic valve with clinical measurements. The study is intended as a proof of concept that such a model can be used to reliably predict maximum pressure in the left ventricles. The reliability and accuracy of this method for clinical use with human subjects would require appropriate clinical studies.

## Abbreviations

MPLV: Maximum pressure in the left ventricle; ALE: Arbitrary Lagrangian–Eulerian; FSI: Fluid–structure interaction; COT: Thermodilution cardiac output; COF: Fick oximetric cardiac output; COD: Doppler cardiac output; VSP: Ventricular systolic pressure; ADP: Aortic diastolic pressure; ADPD: FSI prediction of aortic diastolic pressure’s change relative to heart rate based on Doppler method; ADPF: FSI prediction of aortic diastolic pressure’s change relative to heart rate based on Fick oximetric method; ADPT: FSI prediction of aortic diastolic pressure’s change relative to heart rate based on Thermodilution method; VSPD: FSI prediction of ventricular systolic pressure’s change relative to heart rate based on Doppler method; VSPF: FSI prediction of ventricular systolic pressure’s change relative to heart rate based on Fick oximetric method; VSPT: FSI prediction of ventricular systolic pressure’s change relative to heart rate based on Thermodilution method.

## Competing interests

The authors of the manuscript declare that they have no conflict of interest.

## Authors’ contributions

HGB has done the modeling and simulations, KH, AKH, and DKS reviewed the results, MN, NF approved the final results and discussions, and DME commented the structure of the research, edited the manuscript and revised it. All authors read and approved the final manuscript.
